# Leveraging Antibody, B Cell and Fc Receptor Interactions to Understand Heterogeneous Immune Responses in Tuberculosis

**DOI:** 10.3389/fimmu.2022.830482

**Published:** 2022-03-17

**Authors:** Stephen M. Carpenter, Lenette L. Lu

**Affiliations:** ^1^Division of Infectious Disease and HIV Medicine, Department of Medicine, Case Western Reserve University, Cleveland, OH, United States; ^2^Cleveland Medical Center, University Hospitals Cleveland Medical Center, Cleveland, OH, United States; ^3^Division of Geographic Medicine and Infectious Diseases, Department of Internal Medicine, UT Southwestern Medical Center, Dallas, TX, United States; ^4^Department of Immunology, UT Southwestern Medical Center, Dallas, TX, United States; ^5^Parkland Health and Hospital System, Dallas, TX, United States

**Keywords:** antibody, Fc receptor, B cell, tuberculosis, Fc effector function, iBALT, antigen presentation, T cell

## Abstract

Despite over a century of research, *Mycobacterium tuberculosis (Mtb)*, the causative agent of tuberculosis (TB), continues to kill 1.5 million people annually. Though less than 10% of infected individuals develop active disease, the specific host immune responses that lead to *Mtb* transmission and death, as well as those that are protective, are not yet fully defined. Recent immune correlative studies demonstrate that the spectrum of infection and disease is more heterogenous than has been classically defined. Moreover, emerging translational and animal model data attribute a diverse immune repertoire to TB outcomes. Thus, protective and detrimental immune responses to *Mtb* likely encompass a framework that is broader than T helper type 1 (Th1) immunity. Antibodies, Fc receptor interactions and B cells are underexplored host responses to *Mtb*. Poised at the interface of initial bacterial host interactions and in granulomatous lesions, antibodies and Fc receptors expressed on macrophages, neutrophils, dendritic cells, natural killer cells, T and B cells have the potential to influence local and systemic adaptive immune responses. Broadening the paradigm of protective immunity will offer new paths to improve diagnostics and vaccines to reduce the morbidity and mortality of TB.

## The Challenge of Heterogeneity in Clinical Tuberculosis

Every six seconds, an individual is diagnosed with tuberculosis (TB); every twenty seconds an individual dies from active disease (1). This level of morbidity and mortality persists today despite shorter antimicrobial treatment regimens and more sensitive, specific and widely distributed diagnostics. Strategies that prevent active disease are necessary to complement advances in detection and cure. However, defining the targets of prevention is challenged by the heterogeneity of manifestations and outcomes in clinical TB.

**Figure 1:**
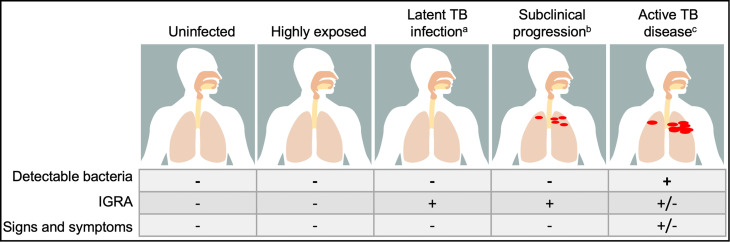
The spectrum of outcomes in human tuberculosis (TB) is heterogenous. Classical clinical states are defines by the presence of detectable bacteria and host response to *Mycobacterium tuberculosis* antigens by interferon *γ* release assay (IGRA), with uninfected having neither, latent TB infection having a positive IGRA but no detectable bacteria and active TB disease diagnosed by the capture of *Mtb* by growth in culture, nucleic acid amplification or cell wall stain. The transition between these states is fluid and poorly captured by these criteria: a. Individuals who have received antibiotic therapy for latent TB infection cannot be differentiated from those who are treatment naïve. b. Individuals who progress subclinically from latent infection to active disease (5-10%) and those who regress or remain asymptomatic (>90%) are indistinguishable. c. After successful treatment with antibiotics for active TB disease, individuals no longer have detectable bacteria as captured by standard assays but may have residual positive IGRA. Moreover, emerging epidemiological and immune correlates data suggest that beyond these classical states, there are groups who are highly exposed to *Mtb* who potentially represent an alternative state to latent TB infection not yet clearly defined.

Improvements in microbiologic, immunologic and radiographic tools to identify and characterize humans infected with *Mycobacterium tuberculosis* (*Mtb*) have expanded the phenotypic spectrum appreciated within and beyond the classical states of latent infection and active disease (**Figure 1**). Over 90% of human TB is thought to exist as latent infection. This state is defined by the presence of IFNγ secreting T cell response to *Mtb* antigens after exposure to the bacteria and the absence of signs and symptoms of active disease. The host response is historically captured by the tuberculin skin test (TST), a cell mediated response to intradermal injection of a mixture of *Mtb* proteins prepared from culture called purified protein derivative (PPD). To overcome false positive responses to non-tuberculous mycobacteria (NTM), including the vaccine strain, *Mycobacterium bovis* Bacille Calmette-Guérin (BCG), IFNγ release assays (IGRA) were developed as blood tests that measure responses to the *Mtb* proteins ESAT6, CFP10 and TB7.7. Yet despite enhanced specificity, the rates of false negatives have limited the use of these T cell-based tests in the diagnosis of the remaining 5-10% of TB which is active disease. Instead, active TB disease is defined by the presence of clinical signs and symptoms, radiographic evidence of disease and microbiological evidence of bacteria (detectable by culture, cell wall stain or nucleic acid amplification). Thus, only latent but not active TB is routinely diagnosed using markers of the host immune response. Specific patterns of human immune reactivity that are sterilizing have not yet been discovered but has significant implications for understanding protection from *Mtb* infection and disease (2).

Beyond latent and active TB, immunological and imaging modalities point towards states of infection after *Mtb* exposure that do not fall into the latent versus active TB dichotomy (**Figure 1**). Waxing and waning T cell and antibody responses to *Mtb* antigens in a subset of individuals with “latent TB” suggest the existence of states after *Mtb* exposure outside of the traditional definition but are also not active disease (3). Detection of *Mtb* RNA and DNA from individuals who meet the definitions of clinical and microbiological cure show bacterial persistence, highlighting the difficulty of conventional immune and microbiological assays to identify the presence of bacteria (4, 5). Finally, highly sensitive computed tomography (CT) and positron emission tomography (PET) imaging has demonstrated different lesions concomitantly regressing and progressing within the same individual, indicating immune response heterogeneity between granulomatous lesions that may underlie clinical outcomes (6–9). These findings advance our understanding of the spectrum of clinical TB, demanding similar innovation in models of disease to expand beyond the current T helper type 1 (Th1)-centric immunological paradigm to capture the diverse spectrum of *Mtb* infection and disease (10–12).

## The Potential of Antibodies and B Cells to Influence Tuberculosis

As a major arm of adaptive immunity, B cells and antibodies have the potential to modulate immune responses to *Mtb*. In contrast to the dominant roles uncovered in responses to viral and other bacterial infections, antibodies and B cells fill a minor part of the literature on the host response to *Mtb*. Yet even in this limited evaluation, there is data to suggest that humoral immunity is a complex and promising path towards understanding TB immunology.

Divergent interpretations of antibody and B cell data reflect limitations in contemporary experimental systems. Ablation of B cell (13) and antibody Fc effector functions (14) in mice and non-human primate models (15) impact local bacterial burden and pathology, and antibody Fc features correlate with different human disease states (16–18). Yet, passive transfer of antibodies into mice do not consistently confer protection (19), human deficiencies in immunoglobulins or B cells do not incur increased risk of TB (20, 21) and antibody titers alone are unable to define infection and disease (22). For animal studies, one of the inherent caveats is that models imprecisely phenocopy human *Mtb* infection and disease. For example, the wildly used inbred C57BL6 mouse model does not form the same granuloma pathology (23) and have different antibody and Fc receptor (FcR) repertoires compared to humans (24). For human studies, power is limited by access to relevant tissues containing *Mtb* and heterogenous clinical manifestations requiring years in duration for evaluation. For studies of humoral immunity, these difficulties are compounded by the persistence of residual antibodies and B cells despite pharmacologic depletion of plasma and B cells in humans (25, 26). As such, the potential of antibodies and B cells to influence the course of TB is inescapably stitched from studies that identify correlates of protection and disease in humans and evaluate mechanisms in model systems. Here, we extrapolate from the literature to understand the capacity of antibodies and B cells to: (1) modulate initial interactions between *Mtb* and host cells, (2) guide the development of adaptive immunity, and (3) contribute to protection and disease across the spectrum of clinical TB.

## Functional Diversity in Antibodies and B Cells

### Antigen Binding Fraction (Fab) Domain

Computational analyses of high throughput sequencing data estimate that the human antibody repertoire has the capacity to bind to 10^13^ unique molecules *via* the antigen binding fragment Fab domain (27). Targeted molecules span proteins, glycans, lipids and nucleic acids from self and non-self, contributing to specific and broad recognition patterns. For *Mtb*, potential antigens recognized by antibodies emanate from the over 4000 open reading frames (28) as well as carbohydrates and lipids that comprise ~60% of the bacterial cell envelope (29). Because of molecular mimicry between microbial and human glyco- and lipo- conjugates, broadly reactive antibodies even at low affinity and avidity have the potential to influence *Mtb*-host interactions. As such, somatic recombination of V(D)J gene segments and hypermutation in the naïve variable gene region following antigen recognition generate diverse Fab domains with the potential to induce different antibody mediated immune responses and clinical outcomes. Yet efforts to develop antibody-based diagnostics demonstrate that titers poorly capture the spectrum of TB (22). Thus, the search continues for *Mtb* antigens that induce relevant immune correlates *via* new combinations of protein, carbohydrate and lipid targets such as lipoarabinomannan (LAM). Moreover, the inability of indirect measures of mycobacterial antigenic burden, such as that by the antibody Fab domain to capture the complexity of *Mtb* infection and disease, compel the pursuit of immune responses beyond direct target detection.

### Crystallizable Fraction (Fc) Domain

**Figure 2:**
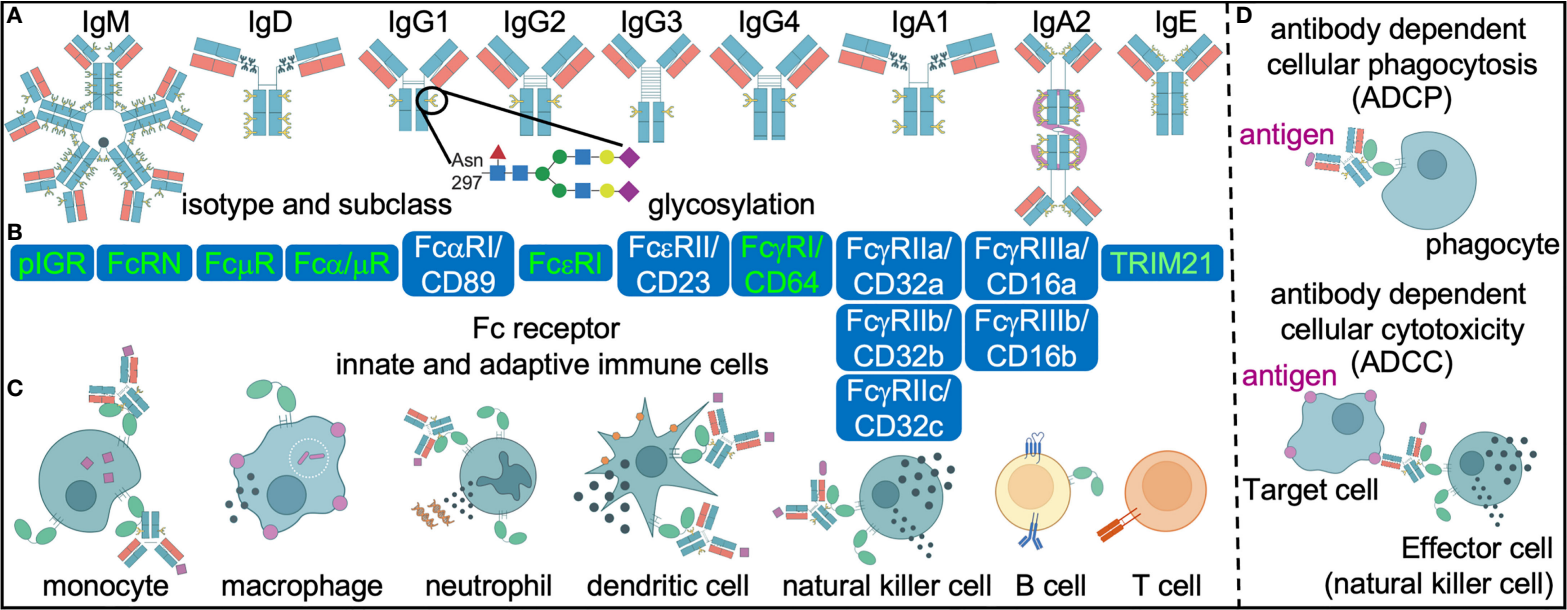
Differential antibody engagement with Fc receptors mediate diverse innate and adaptive immune cell effector functions. Antibody diversity **(A)** in antigen specificity (theoretical n=10^13^), isotype (n=5), subclass (n=6) and post-translational glycosylation (theoretical n=36 possible different moieties) influence engagement of Fc receptors. Subsequent differential activation of high (red) and low (white) affinity Fc receptors **(B)** expressed on innate and adaptive immune cells **(C)** have the potential to mediate effector functions such as antibody dependent cellular phagocytosis and cytotoxicity **(D)** that in concert determine the heterogenous outcomes in human TB.

Antibody function is determined by the combination of the Fab and crystallizable (Fc) domains. The Fc domain can alter the structure of the Fab domain, impacting antigen recognition. Equally as important as antigen binding, the Fc domain is essential to recruiting innate and adaptive humoral and cellular immune responses. Diversity in the Fc domain is generated through different isotypes (IgM, IgG, IgA, IgE, IgD) and subclasses (IgG1, IgG2, IgG3, IgG4, IgA1, IgA2) (**Figure 2A**). Studies of polyclonal responses show that all are produced in response to *Mtb* infection with no one dominating correlate of disease or protection, although there are hints in some cohorts that decreased IgG3 is associated with recurrence (30) and increased IgG4 with active disease (18). Some monoclonal studies have suggested potential differential capacities between IgA, IgG and IgM to induce extra and intracellular host environments that restrict or enhance *Mtb* (31, 32). If these findings are confirmed by studies in polyclonal responses that influence clinical outcomes, the mechanisms likely involve differential immune complexing. With monomeric and multimeric antibodies, the affinity and avidity for microbial antigens in the Fab domain and host receptors and complement in the Fc domain can vary widely.

### Antibody Post Translational Glycosylation

All antibodies are glycoproteins. These post translational modifications are made by glycosyltransferases and glycosidases during trafficking through the endoplasmic reticulum and Golgi apparatus of B cells. Some data suggest that further modification is possible in a B cell independent manner through glycosyltransferases secreted into the circulatory system (33–36). While all polyclonal IgG have a conserved asparagine residue 297 on the Fc domain that is glycosylated, only 15-20% are modified on the Fab domain (37). These N-linked glycan structures have a complex bi-antennary core composed of N acetylglucosamine and mannose residues (**Figure 2A**). The further addition and subtraction of N-acetylglucosamine, galactose, sialic acid and fucose extend the structural and functional heterogeneity of the Fc and Fab domains by altering affinity for FcRs and target antigens. O-linked glycosylation of proteins occurs on functional hydroxyl groups of serine and threonine, but site-specific analyses of O-glycans is challenged by the paucity of efficient proteolytic tools. Nevertheless the plethora of data on N-glycans within IgG show distinct and sometimes plastic glycosylation patterns in many physiological states: male versus female, age, pregnancy, infections such as those with HIV and SARS-CoV2, chronic non-infectious diseases such as diabetes and renal disease, autoimmune diseases and exacerbations (38). In some cases such as diabetes, passive transfer of antibodies into mice of differentially glycosylated IgG and the *in vivo* modification of glycosylation show that altered sialic acid is not merely associative but also contributes to non-immune processes such as glucose intolerance through FcR mediated insulin transport (39, 40).

For latent and active TB, the N-glycan profiles on IgG diverge and vary with antigen specificity (16–18, 41). However, some themes that emerge are that glycans associated with an anti-inflammatory state (galactose and sialic acid) are increased in individuals with latent compared to active TB (16). This is consistent with an overall decreased immune mediated inflammation in latent compared to active TB. In contrast, fucose is decreased in latent compared to active TB. The lack of fucose is generally associated with increased inflammation (42, 43). That afucosylated IgG is enhanced in latent TB indicates that this change is not a biomarker of inflammation but rather potentially reflective of an antibody function. Because studies using polyclonal and monoclonal antibodies show that changes in glycosylation influence FcR affinity and cellular effector functions such as antibody dependent cellular phagocytosis (ADCP) and cytotoxicity, differential modulation of immune responses are implicated for latent and active TB. Afucosylated IgG have increased affinity for FcγRIII/CD16 which mediates antibody dependent cellular cytotoxicity (ADCC). Both are enhanced with IgG purified from latent compared to active TB patients, implicating roles in potential protection for TB (16). If and how antibody glycosylation contributes to immune mediated outcomes in the context of HIV, diabetes and renal disease, the most significant risk factors for TB, remain uncharacterized (44). As biomarkers, direct comparison between *Mtb* reactive-IgG glycans and titers in a small cohort of individuals show that the former has enhanced ability to distinguish between latent and active TB (17). This is likely due to the ability of IgG glycans to capture the complexity of host immune responses that connect to pathology as compared to microbial burden alone by using antibody titers. As mediators of protection, one study showed that passive transfer of non-*Mtb* specific polyclonal human IgG into mice after enzymatic removal of glycans increases bacterial burden (45). Whether this applies to *Mtb* specific IgG and what species of glycans specifically are responsible are not known but could shed light into how differential IgG glycosylation is linked to pathology and protection.

IgA and IgM are also glycosylated, but because far more residues are involved, the deconvolution of these complex post translational modifications lags behind IgG. However, emerging data from the mouse model shows changes in IgM glycans with *Mtb* infection (46). With further definition, these post translational mechanisms of antibody diversification that contribute to the extent of the B cell antibody repertoire can be clarified across the clinical spectrum within and beyond the latent and active TB spectrum.

### Neutralizing and Non-Neutralizing Antibody Functions

For infectious diseases, directly neutralizing and non-neutralizing antibody functions potentially impact microbial infection, replication and immune mediated pathology. Through the Fab domain, antibodies recognize and bind to microbial antigens. This could lead to direct neutralizing activity in which the organism is immediately sequestered after binding. In comparison, non-neutralizing antibody functions involve the Fc domain function to a greater extent. In conjunction with targeting an antigen *via* the Fab domain, the Fc domain engages complement and FcRs on immune cells that induce host responses to the microbe. Non-neutralizing antibody functions include cellular phagocytosis, cytotoxicity, activation (including NETosis and antigen presentation) and cytokine production, each of which could be protective or pathogenic based on the specific context of the microbe and host. Demonstration of consistent antibody-mediated direct neutralization of *Mtb* leading to prevention of infection has been elusive to date with monoclonal antibodies. While *in vitro* studies show different non-neutralizing cell mediated functions with polyclonal IgG isolated from individuals with latent TB, active TB (16), after antibiotic treatment (18), after BCG vaccination (32, 47, 48) and/or high exposure to *Mtb* (41, 49), it is less clear whether they are protective, inert or pathogenic *in vivo*.

### Fc Receptors (FcR)

Beyond the antibody glycoprotein, FcR variations in copy number and single nucleotide polymorphisms (SNPs) generate diversity. These exist for receptors that have high (FcγRI/CD64, FcRN encoded by FCGRT, FcεRI), intermediate (polymeric immunoglobulin receptor pIGR and Fα/μ receptor) and low affinity (FcγRII/CD32, FcγRIII/CD16, FcαRI/CD89, FcεRII/CD23) for monomeric immunoglobulins (**Figure 2B**). High variation in copy number has been observed particularly in the *FCGR* loci. For FcγRII/CD32a, the polymorphism R131H (also described as 167) affects the membrane proximal Ig-like domain of the extracellular region and ablates the ability to bind and phagocytose IgG2 coated particles (50, 51). The R/R131 allotype is associated with more severe outcomes in disseminated meningococcal infection (52) and recurrent bacterial infections (53) in some human populations suggest that these FcR changes could have implications across multiple infectious diseases as well as vaccines. For FcγRIII/CD16a, valine at amino acid 158 (also described as 176) increases the affinity for IgG1 and IgG3 *via* interactions with the lower hinge region whereas phenylalanine in this same position enhances IgG4 binding (54, 55). The functional implications of this variation are most well-described with ADCC, where individuals with 158V/V compared to 158F/F have higher levels of this classic natural killer cell mediated function. Some human studies link these allelic variants to autoimmune diseases (56) and responses to monoclonal antibody therapies in cancer (57), though the extrapolated mechanisms and impacts may be obscured by the complexity of these conditions. For the only inhibitory FcR, a loss of function variant FcγRIIBT232 is associated with protection against severe malaria in an East African population and susceptibility to the autoimmune disease systemic lupus erythematosus in the Caucasian population (58). These functional impacts are conceptualized in the context of non-neutralizing antibody activity. However, studies in mice show that Fc-FcR binding is critical in enhancing monoclonal antibody mediated direct neutralization of pathogens including HIV (59, 60), influenza (61, 62) and SARS-CoV2 (63–66). Thus, FcR variations have the potential to influence directly neutralizing and non-neutralizing antibody functions.

For TB, studies focused on *FCGR* genetic variations show a mixed association with outcomes. Increased *FCGR1A/CD64* copy number in individuals with active disease indicates an association with poorer outcomes (67). Because the high affinity FcγRI/CD64 is also identified in the non-human primate model as a correlate of disease it could be simply a marker of general inflammation, or induce pathology (68). Beyond FcγRI/CD64, higher *FCGR3B* copy number in a subpopulation of Ethiopians is associated with the development of TB in people living with HIV, compared to those with HIV alone (69). The data from these two studies suggest that *FCGR* sequence heterogeneity is involved with inflammation in TB. Studies incorporating a larger clinical spectrum from high *Mtb* exposure to outcomes of antimicrobial treatment and recurrence could further clarify how *FCR* genetic variability impacts *Mtb* infection and disease.

In addition to pathology, data from both mouse models and human studies show that FcRs have the capacity to impart effects that protect the host against bacteria. Knockout of the immunoreceptor tyrosine-based activation motif (ITAM) responsible for activating signaling across FcRs in a low dose aerosol C57BL6 mouse model of *Mtb* infection leads to increased pulmonary *Mtb* burden and pathology along with decreased survival (14). Consistent with these findings, mouse knockout of the only the inhibitory FcγRIIb/CD32b leads to decreased pulmonary *Mtb* burden and pathology (14). Because FcγRIIb/CD32b is the only FcR functionally conserved between mice and humans, these findings could have relevance for translating findings. While *in vitro* blocking antibody studies show that *Mtb*-reactive IgG mediated opsonophagocytosis into human monocytes is FcγRII/CD32 dependent, the blocking antibody clone itself does not distinguish between the activating CD32a or inhibitory CD32b in these cells (47). Overcoming this limitation with FcγRIIb/CD32b specific tools could help clarify each respective role (70, 71). Apart from FcγRII/CD32, levels of the activating FcγRIII/CD16 correlate with latent compared to active TB across multiple cohorts (72). In addition, IgG purified from individuals with latent compared to active TB have higher affinity for FcγRIII/CD16 (16). However, the benefit of the activating FcγRIII/CD16 is likely cell type specific as CD16+ monocytes as opposed to macrophages and natural killer cells may be associated with disease severity (73). Finally, data from *in vitro* whole blood assays with blocking antibodies suggest that decreased bacterial replication mediated by polyclonal IgG from health care workers highly exposed to *Mtb* is in part due to FcγRIII/CD16 together with FcγRII/CD32 (49). Thus, the combinatorial engagement of low affinity inhibitory and activating FcRs (74) by differential immune complexes containing distinct antibody Fc determines signaling (75) contributes to immune response variability that could influence host outcomes.

### Antibody Mediated Cellular Effector Functions

Antigen bound antibodies induce cell surface FcR aggregation and downstream signaling to induce effector functions (76) (**Figure 2C**). Antibody dependent cellular cytotoxicity (ADCC) is classically mediated by natural killer cells and macrophages that express FcγRIII/CD16 (**Figure 2D**). Granules containing perforin and granzyme from activated natural killer effector cells as measured by CD107a and IFNγ are released to mediate cytolysis of target cells with antigens present on the surface and recognized by the antibody. Following binding of IgG immune complexes to platelets, the release of serotonin stored in the granules is thought to contribute to systemic shock (77, 78). For FcεR, aggregation can lead to activation of mast cells, eosinophils and basophils with secretion of vasoactive amines and enzymes to generate allergic responses. Thus, the effect of granule release is dependent on the contents.

In addition to FcR aggregation, internalization of the antigen *via* antibody dependent cellular phagocytosis (ADCP) is an Fc effector function that can occur in neutrophils, monocytes, macrophages and dendritic cells (**Figure 2D**). Neutrophils express FcRs that engage IgG and IgA with phagocytosis (79), production of reactive oxygen species (80) and neutrophil extracellular traps (NETs) (81, 82) as potential consequences. Monocytes and macrophages can respond to IgG, IgA and, sometimes, IgE mediated antigen uptake (62) with autophagy (83) and vesicular trafficking into the lysosome (84) as well as inflammasome activation (85, 86). In contrast, ADCP in dendritic cells induces antigen presentation, cytokine production and maturation (70, 87–89). Thus, FcR crosslinking induce multiple effector immune functions that are cell type specific.

In TB, antibody cellular effector functions have been described to potentially impact bacteria during initial acquisition of infection and in latent and active TB. *In vitro Mtb* infection studies show that opsonophagocytosis is influenced by antibodies from BCG vaccination (32, 48, 90). Because extracellular *Mtb* is thought to exist primarily during initial acquisition of infection and active TB, divergent cellular effector functions resulting from ADCP could be more relevant in these states. Differential macrophage phagolysosomal co-localization and inflammasome activation with intracellular bacteria are reported with polyclonal IgG from latent and active TB (16). Because intracellular *Mtb* is associated with latent infection, cellular effector functions that target an infected host cell as opposed to bacteria alone could be relevant. This could be ADCC but more broadly speaking, any effector cell function that could impact an infected target cell (for example granule or cytokine release, vesicular trafficking, the production of reactive oxygen species) in *cis* or *trans* is plausible. To this point, the discovery of new receptors that bind to antibodies such as the cytoplasmic TRIM21 brings opportunities to expand the repertoire of linked cellular effector functions such as ubiquitination and degradation of intracellular organisms (91). How cytoplasmic receptors, as opposed to cell surface expressing receptors, mediate responses to intracellular *Mtb* is not known but broadens the potential mechanisms by which antibodies modulate the immune response in TB.

### B Cell Functions

**Figure 3:**
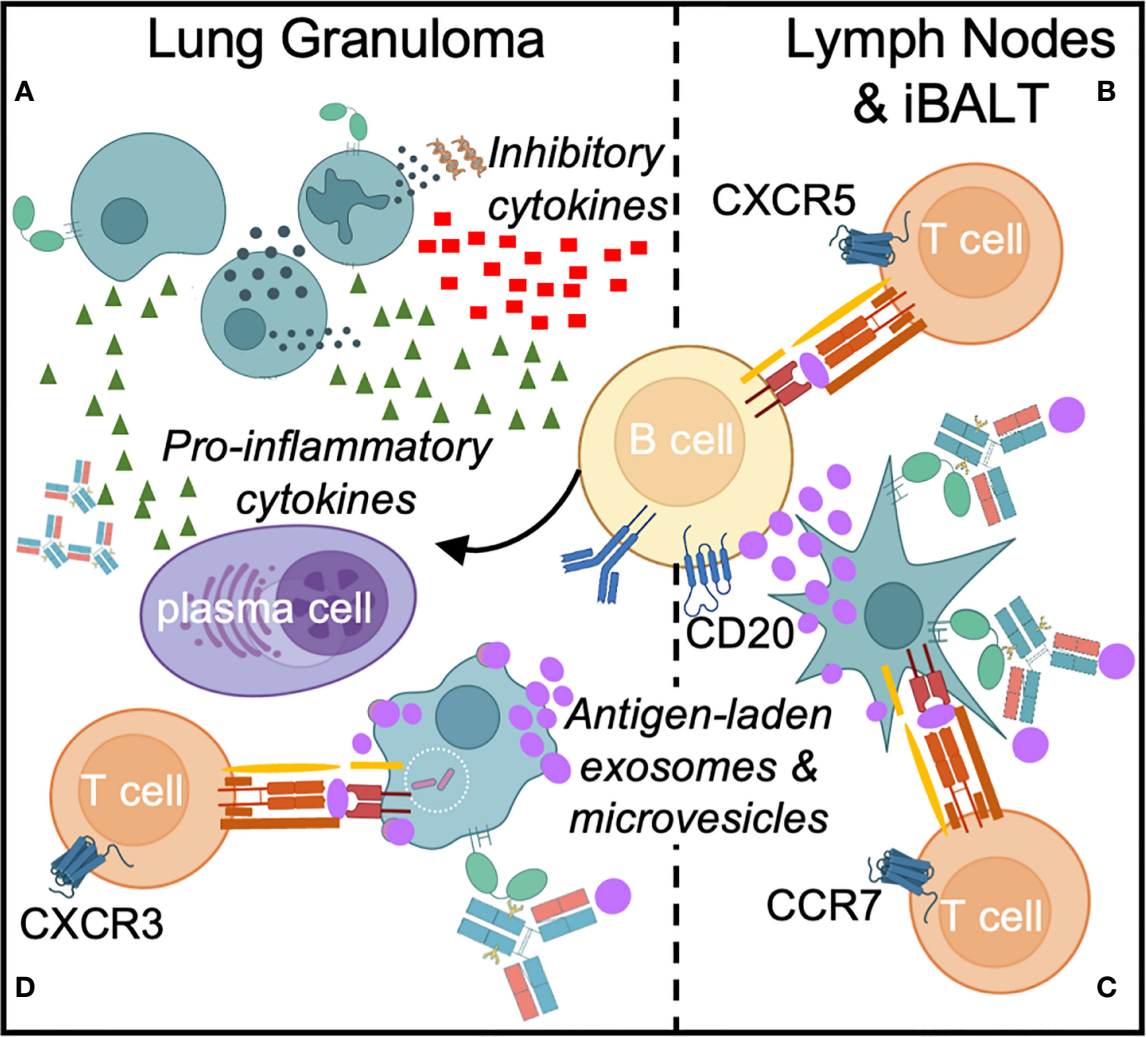
Direct and indirect B cell responses occur in *Mtb* infection. Direct responses include cytokine production and differentiation of B cells specific into plasma cells that secrete *Mtb*-specific antibodies **(A)**. In response to *Mtb* infection in the lung, B cell effectors such as memory B cells, plasmablasts and plasma cells secrete pro-inflammatory cytokines (TNFα, IFNγ, IL2, IL6, GM-CSF and CCL3), while a subset of B cells can secrete inhibitory cytokines, such as IL10, which limits Th17 cell and neutrophil infiltration. In secondary and tertiary lymphoid organs such as lymph nodes and inducible bronchus-associated lymphoid tissue (iBALT), B cells facilitate antigen presentation to T cells **(B, C)**. B cells receive T cell help from Tfh cells to initiate germinal center responses **(B)** and might also participate in priming T cells through the transfer of *Mtb* antigens (purple) to dendritic cells via exosomes and extracellular microvesicles **(C)**. Finally, Fc-FcR interactions can enhance *Mtb* phagocytosis, macrophage and dendritic cell effecror functions, and antigen presentation to T cells in the lung granuloma **(D)**.

T cell-B cell interactions in lymphoid follicles lead to the development of long-lived, antigen-specific humoral responses from plasma cells and plasmablasts (92) (**Figure 3A**). Expression of the CXCR5 chemokine receptor helps define the structure of B cell follicles by facilitating CD4 T cell trafficking to germinal centers. In secondary lymphoid organs such as lymph nodes and tertiary lymphoid structures in the *Mtb*-infected lung, CD4+ CXCR5+ T cells are thought to mediate host protective responses (93, 94). CCR7 regulates the trafficking of B cells toward the T cell zone, where they present endocytosed antigen in the form of peptide fragments loaded onto major histocompatibility complex type II (MHC-II) molecules to CD4 T cells (95). In addition, naïve and central memory CD4 T cells express CCR7, enabling circulation through lymphoid organs and interactions with antigen presenting cells (APCs). CCR7 knockout mice have a decreased ability to control *Mtb* growth after high but not low dose aerosol infection (51, 96). The T cell response is delayed in these knockout mice but whether this is due to a decreased CCR7 mediated B cell trafficking and maturation is less clear. Thus, the co-localization of T cells and B cells in secondary and tertiary lymphoid structures are opportunities for reciprocal interaction and synergy that could influence outcomes in TB (**Figures 3B, C**).

The presence of B cells and follicular structures in granulomas and inducible broncho-alveolar associated lymphoid tissue (iBALT) is described in humans, mouse and non-human primate models of TB. These pulmonary lesions are associated with protection against active disease in the non-human primate model (15, 93, 97–99). Furthermore, the identification of *Mtb*-specific antibody responses and their association with protection in latent infection and in other heavily exposed individuals indicate a protective role for B cells in TB (16, 41, 49), potentially through the activation of *Mtb*-specific T cell responses and regulation of inflammation (13).

While dendritic cells classically act as APCs to activate T cells in lymphoid organs, early experiments identified a role for B cells in the priming of antigen-specific CD4 T cell responses to vaccine antigens in mice (100). B cell depletion prior to vaccination ablates subsequent T cell proliferation upon *ex vivo* exposure to APCs and antigen. Interestingly, subcutaneous B cell supplementation proximal to draining lymph nodes at the time of vaccination rescues this muted T cell expansion. Moreover, B cells produce cytokines that regulate T cell responses during infection and persist as antigen-specific memory cells after the resolution of inflammation (101). While patients with active TB have dysfunctional circulating B cells (102–104), the implications for effector functions beyond antibody production and how they impact local (13, 15, 97) and systemic responses remains to be clarified. Because B cell depletion by anti-CD20 antibodies alters T cell activation, cytokine production and *Mtb* burden in pulmonary lesions (15), evaluation at the lesional level may afford the best appreciation of the diverse spectrum of B cell functions.

B cells were only recently observed to act as natural APCs for T cells, promoting their activation and proliferation when B cells were exposed to T follicular helper (Tfh) cell CD40L and IL4 signaling in contrast to IL21 signaling (105, 106). Colocalization at the T cell-B cell border of lymph nodes or within B cell follicles represent opportunities for B cell antigen presentation to T cells, facilitating T helper functions, B cell activation and clonal expansion. Furthermore, the distinct presence of B cell follicles and germinal centers in the lung as a feature of both granulomas and the adjacent iBALT raises the possibility of roles for B cells in local antigen presentation to T cells, either directly or *via* exosomes and microvesicles (107) (**Figures 3C, D**). CD40-activated B cells have been shown to prime at least transient effector CD8 T cell responses to intracellular bacteria such as *Listeria monocytogenes* (108). Dissecting the impact of B cell mediated antigen presentation for the intracellular *Mtb* could provide a path towards recognizing alternative mechanisms of T cell activation.

B cells and plasma cells produce pro-inflammatory cytokines (IFNγ, TNFα, IL2, IL6, CCL3, GM-CSF) that are balanced by the ability of certain subsets to produce anti-inflammatory IL10 and IL17 to regulate T cell responses (109). Exploitation of IL10 associated inhibitory properties by intracellular pathogens have been described with *Salmonella typhimurium* (110) and *Listeria* (111). In mouse models of these infections, stimulation of TLR2 and TLR4 induces IL10 production from B cells which, when inhibited, improves host control of bacterial burden. In non-human primate models of TB, IL10 is thought to regulate local immune responses to *Mtb* in lung granulomas where both T and B cells traffick (112). Thus, it is plausible that like *Salmonella* and *Listeria*, TLR2 signaling induced by *Mtb* (113) could modulate IL10 levels through B cells as well as macrophages to locally influence outcomes in TB lesions (114). Similarly, Th17 responses not only modulate B cell function by inducing class switching but B cells, particularly the regulatory subset, can in turn suppress Th17 responses (115, 116). In patients with more severe cavitary TB disease, CD19+ CD1d+ CD5+ B regulatory cells are increased, dampening IL17 (117, 118). Thus, IL10 and IL17 are additional conduits through which B cells can regulate T cell responses and subsequent protection as well as pathology.

## At the Interface of Innate Host *Mtb* Interactions

### Antibodies in the Respiratory Tract

Because antibodies are detected at the alveolar epithelia, the site of initial *Mtb* exposure in aerosol transmission, they are well-positioned to mediate the initial interactions with bacteria. In healthy humans, bronchial sampling by lavage demonstrates that IgG is present in the respiratory tract at levels equivalent to peripheral serum (119). During infection by the SARS-CoV2 virus, IgG levels increase even beyond those of IgA (120). In the widely used C57BL6 mouse model, IgG, IgA, and IgM are detected in the uninfected lung parenchyma with IgG2b levels in bronchoalveolar lavage (BAL) fluid higher than those of IgA (121). Thus, multiple isotypes and subclasses are likely present in the respiratory tract for these early events.

### Cross Reactive and Specific Antibodies That Recognize *Mtb*

The repertoire of *Mtb* antigens recognized by antibodies in the respiratory tract, as well as systemically, remains a critical gap in knowledge. However, BCG vaccination (32, 47, 48, 122) and exposure to environmentally prevalent non-tuberculous mycobacteria likely introduce at minimum cell wall antigens that elicit antibodies cross-reactive to *Mtb* (32, 123, 124). As in the gut, symbiotic microbiota from the pulmonary compartment could induce systemic IgG responses that induce protection against pathogens (125). These antibodies arising from non-self antigens complement those from self. Natural IgM and IgG have the ability to engage with carbohydrate and lipid *Mtb* targets to influence initial host-microbial interactions and subsequent infectious challenge (126). As the products of B-1 cells (127) that arise during immune development independent of exogenous antigens and T cells, natural IgM targeting self-antigens such as phosphorylcholine modulate autoimmunity and responses to virus *via* antibody mediated induction of complement (128) and efferocytosis of apoptotic cells (129, 130). In murine influenza studies, passive transfer of IgM from uninfected mice into those deficient in B-1 B cells or are unable to secrete IgM enhance survival and microbial specific IgG levels, suggestive of a role in modulating the development of the adaptive immune response in secondary lymphoid organs (131, 132). Beyond IgM, data from mouse studies suggest that natural IgG can aid in FcR mediated phagocytosis of ficolin coated gram negative and positive bacteria to impact susceptibility to infection (133, 134). Whether by specific and or cross-reactive antigen recognition, these studies suggest that localized and systemic antibodies are poised to recognize *Mtb* and mediate initial interactions within even an uninfected, naïve host.

In individuals who have developed an adaptive immune response to *Mtb* infection, the over 4000 open reading frames and the plethora of cell membrane and wall lipids and glycolipids provide a spectrum of *Mtb* targets to which antibodies can direct immune responses (28, 29). Because replicative and non-replicative *Mtb* states vary in relative abundance of these microbial antigens (135, 136), different specificities may be relevant for antibody functions in latent TB, active TB and with prior antimicrobial treatment. T cell responses to genes encoded by the dormancy regulon of *Mtb* are enhanced in latent infection (137) but the impact from the antibody standpoint is not known. Moreover, different clinical strains could mean that the Fab domain repertoire vary by geographical region (138). It is presumed that the majority of *Mtb* targets are expressed by live bacteria and infected host cells including epithelial cells (139) as well as macrophages (140). However, exosomes in the blood and granuloma also contain *Mtb* antigens that can activate immune cells *in vitro* and *in vivo* (141, 142). Thus, antigen specific antibody effector functions can be induced even in the absence of live *Mtb*.

### FcRs in the Respiratory Epithelia

With aerosol transmission, the first encounter between *Mtb* and host likely occur with non-immune cells in the respiratory tract. At this earliest stage, the conditions that determine if the bacteria infect and cause disease are not known. Antibody Fc domain engagement of receptors aid in translocation across the lumen and induce non-immune cells to secrete local cytokines, activating resident immune cells such as alveolar macrophages. Thus, FcR on epithelial airway cells could be the first to encounter antibody-complexed *Mtb* to enable bacterial movement and initiate host responses. The two most abundant FcR in the respiratory tract are the neonatal FcR (FcRN) and polymeric immunoglobulin receptor (pIGR) though other FcRs that bind IgG, IgA and IgE are also expressed.

In the respiratory tract, FcRN facilitates transport of IgG across the mucosal surface (143). Its high affinity nature permits binding to monomeric IgG to guide through the endocytic excretion pathway and extend serum half-life by preventing lysosomal degradation. After low dose aerosol *Mtb* challenge, pulmonary bacterial burden is lower in FcRN knockout compared to wildtype mice at early but not late timepoints. However, there is an absence of differences in initial inoculum (121) and dissemination into the spleen and liver. These findings indicate that though uptake may be similar *via* non-*Mtb* specific antibodies, subsequent host responses and bacterial outcomes are transiently affected (121). Monoclonal *Mtb* specific IgG but not IgA increases opsinophagocytosis in FcRN expressing A549 human lung epithelial cell line (31). Thus, *in vivo* and *in vitro* data suggest that *Mtb* reactive IgG from prior mycobacterial exposure enhances early infection transiently *via* FcRN at the epithelium. The implications for outcomes in chronic disease and in the presence of *Mtb* reactive antibodies induced by BCG vaccination, prior *Mtb* or NTM infection are less clear but likely involve immune as well as epithelial cells.

Similar to FcRN, the polymeric immunoglobulin receptor (pIGR) is widely expressed in the respiratory tract. Unlike FcRN which focuses on IgG, pIGR supports the transport of dimeric IgA and, to a lesser extent, IgM from the interior basolateral surface of the epithelium to the exterior apical side. Interestingly, IgA levels are decreased in saliva but not bronchoalveolar lavage in a total mouse pIGR knockout (144). In these mice, there is an early and unsustained increase in pulmonary bacterial burden and decrease in IFNγ and TNFα after low dose aerosol infection with virulent *Mtb* strain H37Rv. These data suggest that the effects of non-specific IgA transported to upper respiratory tract by pIGR can be transiently protective in early infection.

Direct neutralization by *Mtb* specific compared to non-specific IgA in the mucosa that blocks bacterial uptake could mediate a more significant and durable effect. Monoclonal antibodies targeting the mycobacterial glycosylated lipoprotein phosphate transporter subunit PstS1 demonstrate this potential in human lung epithelial cells *in vitro* (31). Polyclonal antibodies induced after intranasal vaccination with PstS1 shows that *in vivo* protection in a high dose intranasal BCG challenge model can be generated in a pIGR dependent manner (31). That FcαR/CD89, the primary human IgA FcR, is not detected on human epithelial cells or expressed in mice points towards direct neutralization as one likely mechanism. However, other receptors for IgA including Fcα/μ receptors (145), asialoglycoprotein receptors (146), transferrin receptors (147) and M cell receptors (145, 148) have been reported to be able to bind to the IgA Fc domain. These receptors provide unexplored FcαR/CD89 independent mechanisms of non-neutralization by which *Mtb* specific IgA that could inhibit bacteria in the respiratory tract.

While the type I FcγRs are commonly described in cells of hematopoietic lineage, which consequently have been the focus of most functional studies, some data suggest that these FcRs can be found in non-immune cells, permitting IgG to induce non-neutralizing functions. The low affinity FcγRIII/CD16 is expressed in primary nasal epithelial cells and *in vitro* blocking antibody studies demonstrate a role in mediating IgG opsonized bacterial cytokine induction (149). The low affinity inhibitory FcγRIIb/CD32b but not the IgE receptor FcεRIII/CD23 is present in primary human airway smooth muscle cells (150). Understanding non-neutralizing Fc effector functions mediated by non-immune cells in the airway lumen to *Mtb* could provide insight into the factors that determine whether colonization or deeper infection occurs.

### FcRs Mediating Macrophage Environments That Permit and Restrict *Mtb* Growth

Macrophages represent a quintessential niche for *Mtb* where bacteria grow and die. In the high dose aerosol murine infection model, tissue resident, long-lived alveolar macrophages act as initial host cell targets for *Mtb* (151) and contribute to the development of immune conditions that permit *Mtb* replication and dissemination (151, 152). Bone marrow derived monocytes and macrophages are subsequently recruited to join their embryonic tissue resident counterparts. Further infection of these populations show heterogeneity between and within the groups. Initial studies suggested an M1 and M2 paradigm to describe macrophage restrictiveness and permissiveness to *Mtb* (153). However, more recent data from single cell mouse and human macrophage transcriptomics suggest that the determinants of bacterial outcomes are far more complex (154, 155). Whether or not FcR mediated signaling contribute to the heterogeneity of macrophage responses to *Mtb* is not known, but the combinatorial diversity from engagement of the multiple low and high affinity receptors expressed in this immune cell provides this potential.

Macrophages express a plethora of FcRs that enable responsiveness to antibodies found in the blood and tissue, including *Mtb* lesions such as granulomas (156). High affinity FcγRI/CD64 and FcRN allow monomeric IgG to influence macrophage phenotypes. The low affinity activating FcγRIIIa/CD16a and FcγRIIa/CD32a permit further tuning with IgG immune complexes, counterbalanced by the only inhibitory receptor FcγRIIb/CD32b. In being the only immune cell other than natural killer cells that expresses a significant level of FcγRIIIa/CD16a which classically mediates ADCC, macrophages have the potential to be both effector and target cells. In being like all other immune cells where the cytosolic FcR TRIM21 is expressed, antibodies may also enable the macrophage to target intracellular *Mtb*. For tissue macrophages including alveolar macrophages the activating low affinity FcγRIIIa/CD16 and high affinity FcγRI/CD64 are particularly highly expressed when compared to blood monocyte derived macrophages (157, 158). Examining the link between FcRs and cell population specific responses to *Mtb* could clarify how macrophages permit and restrict bacterial replication.

Classic experiments show that extracellular *Mtb* uptake mediated by live BCG immunized rabbit serum into mouse peritoneal macrophages direct bacteria into phagosomes, though the outcomes for the bacteria and host vary (159). Trafficking of *Mtb* into the phagolysomal and autophagosomal compartments, induction of pro- and anti- inflammatory cytokines and likely multiple other cellular effector functions collaboratively determine intracellular bacterial fate. Much of the literature has focused on opsonophagocytosis. Thus, classical FcRs at the cell surface involved in ADCP including FcγRI/CD64, FcγRII/CD32 and, to a significantly lesser extent, FcγRIII/CD16a (47) play roles in uptake of extracellular *Mtb* into monocytes and likely macrophages. For the intracellular bacteria *Legionella* (160), the initial steps of FcR mediated entry into the macrophage govern intracellular fate, but for *Mtb* this remains unknown.

As important as extracellular *Mtb*, *in vitro* data suggest that antibodies can also impact intracellular bacteria, the predominant state in infection. Addition of polyclonal IgG from latent compared to active TB patients to macrophages already infected with *Mtb* induce lower intracellular burden (16). That these antibodies have higher affinity for FcγRIIIa/CD16a and induce higher ADCC *in vitro* with natural killer cells that express FcγRIIIa/CD16a suggests that a similar mechanism could be occurring with *Mtb* infected macrophages as target and effector cells. An alternative and complementary mechanism could be *via* the cytosolic E3 ubiquitin ligase TRIM21. This FcR is able to ubiquitinate antibody bound intracellular viruses and bacteria such as *Salmonella* for degradation (91), though for *Mtb* this is not yet known. Thus, antibodies through surface and cytoplasmic FcRs can impact intracellular *Mtb* directly or indirectly to restrict growth.

Whether antibodies induce macrophages to restrict or permit *Mtb* infection and replication likely results from the sum of engaging multiple high and low affinity activating and inhibitory FcRs, which could differ for extracellular and intracellular bacteria. Additional immune cell signaling pathways could also contribute. For example, *Mtb* components interact with Toll like receptors (TLR) (161) to induce innate macrophage responses. Cross talk between TLR and FcR signals on macrophages have been described (162). Understanding the interactions of these pathways for *Mtb* could highlight macrophage diversity in the context of bacteria, antibodies and pathogen associated molecular patterns (PAMPs) that exist in concert throughout infection and disease.

### FcR Mediated Neutrophil Inflammation in TB

Like in macrophages, much of the literature on neutrophils in TB have focused on inflammation in active TB (3, 163). Correlations between blood transcriptional signatures with disease suggest that neutrophils mediate or reflect pathogenic inflammation. While expression levels of the high affinity FcγRI/CD64 is consistent with this, additional low affinity receptors capable of binding to IgG and IgA provide the potential for antibody mediated protective effector functions *via* the peripheral blood and granuloma where neutrophils surround *Mtb* infected macrophages.

Neutrophils have a partially overlapping FcR repertoire with macrophages. Like macrophages, the low affinity activating FcγRIIa/CD32a, and inhibitory FcγRIIIb/CD32b, as well as high affinity FcRN and TRIM21 are constitutively expressed. Unlike macrophages, high FcαR/CD89 expression characterizes these granulocytes, permitting responses to IgA. The monomeric isoform FcγRIIIb/CD16b is constitutively expressed instead of the heterooligomeric FcγRIIIa/CD16a. FcγRI/CD64 expression is not constitutive but rather induced by IFNγ (164). Thus, baseline and induced FcR permit a spectrum of neutrophil phenotypes in *Mtb* infection.

Transcriptomics data show that FcγRI/CD64 expression and neutrophil activation are correlates of TB disease progression across multiple human cohorts (3, 165) and mouse and non-human primate models (68). However, despite bearing the same names, murine and human CD64 differ in their binding abilities and expression patterns such that they are not functional homologues. Knockout in mice leads to a transient decrease in pulmonary *Mtb* at 60 days along with decreased neutrophil but not macrophage recruitment to the lungs, suggesting that FcγRI/CD64 enhances disease (68). Though there are limitations to extrapolating these mouse data to humans, increased *FCGR1/CD64* copy number in individuals with active disease shows association with poorer outcomes (67). Neutrophil FcγRI/CD64 expression is a biomarker for sepsis in children and adults with bacterial infections (166). As such, FcγRI/CD64 is likely similarly a correlate of inflammation for TB.

In contrast, the expression of FcγRIIa/CD32a and FcγRIIIb/CD16b on neutrophils suggests the potential existence of an IgG mediated protective function. While ADCC is typically described as a natural killer cell FcγRIIIa/CD16a function, neutrophils can also be effector cells with FcγRIIa/CD32a being the activating receptor, negatively regulated by FcγRIIIb/CD16b as shown in tumor models (167). In TB, higher *FCGR3B* copy number in a subpopulation of Ethiopians is associated with HIV-TB compared to HIV alone (69), suggesting that decreased neutrophil ADCC could be associated with more severe disease in dually infected individuals.

Beyond IgG, studies using human FcαR CD89 transgenic mice point towards the potential for IgA mediated FcR functions to impact *Mtb*. Opsonization of H37Rv with human monoclonal IgA targeting the alpha crystallin *Mtb* protein HspX prior to high dose intranasal challenge of these mice leads to decreased pulmonary bacterial burden early after infection compared to non-transgenic littermates. Thus, beyond direct binding, IgA Fc mediated non-neutralizing functions through FcαR/CD89 can impact events that affect the development of adaptive immunity (168). Whether protection occurs *via* neutrophils, monocytes/macrophages or other immune cells, the durability of such protection and how much can be translated into humans remain to be clarified (169). Intriguingly, IgA monoclonals can also induce neutrophil-mediated ADCC (170), providing a plausible mechanism by which antibodies can inhibit *Mtb*. In contrast, there is evidence that both IgA and IgG can induce neutrophil and monocyte mediated trogocytosis (171, 172), a phagocyte nibbling process which can contribute to the spread of intracellular organisms such as *Francisella tularensis* and *Salmonella* from infected to uninfected cells (173). Thus, some antibody induced neutrophil functions could contribute to *Mtb* pathogenesis. Indeed, NETosis is initiated by many receptors, amongst which FcγR (174, 175) and FcαR (81) are members. As NET formation is detected in necrotic lung lesions of TB patients which promote bacterial growth (163), antibody mediated neutrophil activation can be as locally pathologic as it is systemically.

### FcRs Influencing Dendritic Cell Antigen Presentation

Dendritic cells are the most efficient professional APCs that prime T cells. As such, enhancement of dendritic cell functions in the context of vaccination can in animal models produce sterilizing protection for *Mtb* (176). Studies in other infectious disease and cancer models show that antibodies through FcR on dendritic cells bridge the gap between innate and adaptive responses, but how this can confer long lasting immune memory for TB in a physiologically relevant setting continues to be re-evaluated.

Similar to macrophages, dendritic cells express a plethora of receptors that permit responsiveness to antibodies in the lung upon recruitment in *Mtb* infection. Monocyte derived dendritic cells and macrophages express baseline high levels of activating FcγRs, and conventional and plasmacytoid dendritic cells can also express the only inhibitory FcγR FcγRIIb/CD32b (177). By engaging both activating FcγRIIa/CD32a and inhibitory FcγRIIb/CD32b low affinity signaling, IgG can influence antigen uptake and presentation, maturation and cytokine production. These activities likely direct priming either at the primary pulmonary site of *Mtb* infection or in the draining lymph nodes as adaptive immunity develops (70, 87–89, 178). Moreover, in addition to its recycling function, the high affinity FcRN in concert with FcγRIIa/CD32a can regulate cross presentation of IgG immune complexes (179). Exactly how this occurs in TB is unclear. FcRN knockout mice have enhanced *Mtb* infected CD103+ dendritic cells and CD4 T cell priming early at day 14 of infection, with decreased *Mtb* burden on days 14 and 28. However, it appears that this effect is transient, leading to similar pulmonary burdens on day 56 (121).

An anti-tumor T cell “vaccinal effect” through engagement of FcγRIIa/CD32a on dendritic cells can be induced in a human *FCGR* transgenic mouse model with monoclonal antibodies. This is in addition to the transient clearance of tumor cell targets by macrophage FcγRIIIa/CD16a mediated ADCC (180). This FcγRIIa/CD32a dendritic cell mediated effect is even more pronounced in the context of influenza infection. Fc engineered antibodies show that binding to FcγRIIa/CD32a on dendritic cells increases CD40, CD80 and CD86 expression that induces protective CD8 T cell activation while FcgRIIIa/CD16a on macrophages had a limited role (181). A direct link between antibody mediated effects on dendritic cell antigen presentation and B and T cell maturation have yet to be shown for *Mtb*. However, any protective *in vitro* effect of polyclonal IgG on dendritic cells from humans highly exposed to *Mtb* is dependent on the presence of MHC-II and CD4 T cells in whole blood (49). This suggests that the interaction is plausible in natural exposure and could be leveraged by vaccines.

### Regulation by the Inhibitory FcγRIIb/CD32b

Activating FcR mediated immune responses in the absence of checks and balances lead to autoimmune induced pathology. Though there are multiple regulatory points, FcγRIIb/CD32b is the only inhibitory FcγR and is the only FcγR well conserved between humans and mice (182). The high sequence similarity between the extracellular domains of *FCGRIIB/CD32B*, *FCGRIIA/CD32A* and *FCGRIIC/CD32C* and the paucity of specific monoclonal antibody probes limit experimental designs. However, the knockout mouse model has provided some hints as to the importance of inhibitory FcγR regulation in TB.

In response to low dose *Mtb* aerosol infection, mice genetically deficient in FcγRIIb/CD32b have increased CD4 T cell IFNγ production, decreased bacterial burden in the lungs and spleen, and decreased neutrophilic inflammation at day 30 (13). Thus, in early infection, activating FcR signaling that enhance CD4 T cells are protective. However, the implications for chronic disease, which is more characteristic of human TB, are unclear as the autoimmune phenotype of these knockout mice confound the interpretation of *Mtb* survival experiments that last over one year.

Because FcγRIIb/CD32b is detectable on dendritic cells and subpopulations of monocytes, macrophages and neutrophils, it is likely that the cumulative impact of the only inhibitory FcγR for TB reflects functions from all of these immune cells. In human (70) and mouse dendritic cells (88), FcγRIIb/CD32b counter balances FcγRIIa/CD32a activation of maturation, secretion of inflammatory cytokines and MHC-I and MHC-II antigen presentation (87). Thus, loss of FcγRIIb/CD32b in dendritic cells likely promotes the development of a Th1 response able to inhibit *Mtb*. However, FcγRIIb/CD32b in follicular dendritic cells counterintuitively enhances B cell activation by multimerizing antigens to crosslink multiple B cell receptors (BCRs) (183). The existence of this T cell independent mechanism of antibody induction suggests stark differences within dendritic cell subsets that could have implications for long lasting immunity. For monocytes, macrophages and other granulocytes, the presence of activating FcRs determine the impact of FcγRIIb/CD32b (74, 75, 156). This further argues that FcγRIIb/CD32b functions are likely highlighted primarily in the context of stimulatory factors as opposed to acting in isolation.

In B cells, FcγRIIb/CD32b negatively regulates BCR signaling, expansion and plasma cell differentiation (184), suggesting that antibody production is also inhibited. Thus, *Mtb* specific antibodies generated in knockout mice could be higher when compared to wildtype. Higher levels of IgG, IgM and IgA could enhance immune cell effector functions, further contributing to anti-*Mtb* activities in the absence of an inhibitory checkpoint.

Finally, there is emerging evidence from adoptive transfer experiments that FcγRIIb/CD32b can act as a CD8 T cell checkpoint inhibitor involved in anti-tumor immunity (185). Extrapolation for *Mtb* would implicate an involvement of the inhibitory FcR more directly in the regulation of cytotoxic T cell functions that could be protective.

## Influencing Adaptive Immunity

### Fc Receptors on T Cells

While MHC-I and MHC-II antigen presentation and expression of co-stimulatory ligands by APCs and B cells represent the primary paths through which CD4 and CD8 T cells are activated, there is some data to suggest that activating FcR can more directly influence T cells. T cell activation and differentiation has been associated with expression of the low affinity FcR, FcγRII/CD32, including both CD4 (186–188) and CD8 T cells (181, 189). On a small subset of CD4 T cells from blood and lymphoid tissues of humans and Rhesus macaques with and without HIV/SIV infection, proliferation, differentiation and cytokine production could be enhanced by immune complex activation of FcγRIIa/CD32a (186, 187). Interestingly, IgM binding to the Fcμ receptor on peripheral human T cells increases T cell receptor (TCR) and CD28 coreceptor expression, thereby lowering the threshold for T cell activation (190). Thus, high levels of IgM such as that induced by intravenous BCG could provide co-stimulation through FcR binding and enhance early T cell responses to lead to protection against *Mtb* (32). Evaluation of these T cell subsets in TB would provide greater clarity as to whether FcR binding could serve a co-stimulatory function *in vivo* when T cells are activated naturally through TCR-dependent mechanisms.

### B Cells in Antigen Presentation to T Cells

The interactions between naïve T cells and APCs within the T cell zones of secondary lymphoid organs determine the repertoire of TCRs, which is governed by antigen availability and TCR binding characteristics (191). B cells recognize and respond to the structure of 3-dimensional antigens, including the simultaneous binding of non-sequential, distant residues which become juxtaposed upon protein folding. However, T cells recognize sequential amino acids within short peptides only when they are presented in the context of the MHC molecule. Since antigens are bound to the BCR, endocytosed, processed and presented as peptides, antibodies produced by differentiated plasma cells might affect the activation of peptide-specific T cells targeting the same *Mtb* protein. Thus, B cells could present antigens for the purpose of activating conventional T cells in a manner similar to their presentation of antigens to T follicular helper (Tfh) cells (**Figure 3B**) (111).

The most well-characterized interactions between B cells and T cells occurs in the context of B cell antigen presentation to Tfh cells in secondary lymphoid organs. This facilitates B cell activation and differentiation into antibody-producing plasma cells. However, the professional antigen-presenting capacity of B cells together with their strategic localization in and around sites of T cell priming in secondary lymphoid organs, granulomas and iBALT after *Mtb* infection suggests a broader role, including participation in *Mtb*-specific T cell activation.

Classically, CD4 T cell priming in response to *Mtb* has been shown to occur when T cells are activated by conventional dendritic cells in lymph nodes after CCR2+ monocyte-derived APCs transport live *Mtb* from the lung to the draining lymph nodes (178, 192, 193). After priming, effector T cells traffick to the lungs and secrete IFNγ and cytokines in response to *Mtb*. However, depletion studies in animal models identified a role for B cell antigen presentation in supporting optimal T cell responses in infectious disease and autoimmune models, suggesting that this too could occur in the context of TB. In a mouse model of adjuvanted peptide vaccination, B cell presentation of peptides on MHC-II was found to be necessary to enhance CD4 T cell expansion and IL2 production (106). B cell depletion using an anti-CD20 monoclonal antibody in mice significantly reduces total baseline numbers of naïve, effector, and regulatory CD4 and CD8 T cells. In the context of lymphocytic choriomeningitis virus (LCMV) Armstrong infection, this leads to increased viral load, an effect which is rescued by infusion of LCMV-specific TCR transgenic CD8 T cells (194). Similarly, B cell depletion prior to infection with *Trypanosoma cruzi* reduces subsequent induction of total and parasite-specific CD8 T cells (195), and B cells are critical in the development of antigen-specific effector and memory CD4 T cell responses to *Listeria* (196, 197) and *Salmonella* (198). Finally, at least two studies have shown a supporting role for B cells in priming autoreactive T cell responses in mouse models of autoimmune diabetes, arthritis and lupus independent of plasma cell differentiation and antibody production (197, 199). These data indicate that B cells assist antimicrobial and autoimmune T cell responses either directly or indirectly.

An indirect mechanism of antigen presentation to T cells by B cells is secretion of MHC-II containing exosomes or extracellular vesicles. In this scenario B cells activate T cells in an HLA-DR dependent manner (200–202). T cell stimulation by extracellular vesicles is dependent on expression of the costimulatory receptor CD28 (203). This mechanism of antigen presentation is of particular interest in TB as both extracellular microvesicles and exosomes from *Mtb* infected macrophages and dendritic cells are sources of antigen that can directly or indirectly activate antigen-specific CD4 and CD8 T cells (204–208). Though not yet studied in TB, B cell exosomes may serve a similar function to provide alternative ways to activate T cells to compensate for Mtb immune evasion in macrophages (209–211). It is also possible that B cell exosomes provide a means of transferring units of MHC class II-peptide complexes between different cells (212) (**Figure 3C**). Thus, MHC-II provides multiple lines of B cell-T cell communications.

In addition to T and B cells, tingible body macrophages (TBMs) are found in the germinal centers in lymph nodes, spleen and iBALT from the non-human primate model of TB (213). TBMs are specialized macrophages that engulf apoptotic B cells during affinity maturation (214, 215). In this manner, TBMs prevent the loading of self-antigens from cell debris immune complexes onto follicular dendritic cells that present to B cells (216, 217). Moreover, studies using ovalbumin-specific T cell hybridomas show that antigen-laden TBMs suppress the ability of B cells to activate T cells (215), thereby preventing autoimmune responses. In the mouse model of TB, dendritic cells uptake extracellular vesicles from apoptotic macrophages infected with *Mtb* and stimulate CD8 T cell responses in an MHC-I or CD1-dependent manner (218). These data imply a role for scavenger APCs in presenting *Mtb* antigens to regulate T cell responses. Cross-priming of CD8 T cells from APCs is likely important for the recognition and killing of many pathogen infected cells. In TB, acquisition of antigens *via* efferocytosis and uptake of extracellular vesicles enhances the number of antigen specific CD8 T cells that recognize *Mtb* infected macrophages (130, 219, 220). TBM mediated efferocytosis and T cell cross-priming could explain the association of B cell follicles with protection against active disease in the non-human primate model of TB (213).

### B Cell Follicle-Like Structures

Like a granuloma, the iBALT is a tertiary lymphoid structures that enrich and facilitate interaction between myeloid cells, B cells, T cells and non-hematopoietic cells (107, 221). Adjacent to granulomas, the iBALT is a pulmonary structure consisting of B cell follicles and components of the lymph node including high endothelial venules and connection the lymphatic drainage (93, 99, 107, 222–225). Induced early after infection, the follicles contain IgD+ B cells clustered around a network of follicular dendritic cells. These stromal cells produce IL1α, express the lymphotoxin α (LTα) receptor and secrete CXCL13 (225) to help recruit immune cells to form the iBALT (226). The iBALT is associated with protection in the setting of *Mtb*, serving as both a lymphoid organ and a lung-resident source of locally-activated, antigen-specific T and B cells primed for rapid responses (107, 213).

The iBALT is a feature of type 1 immune responses and is reported to regulate type 2 and type 17 responses. A recent study in a mouse model of asthma found that pre-existing iBALT delayed the onset of Th2 trafficking and related inflammation (227). In a mouse model of influenza infection, the iBALT offers protection through supporting antigen specific CD8 T cells, humoral responses and the formation of immunological memory even in the absence of spleen and lymph nodes (228, 229). The iBALT has also been shown to recruit dendritic cells harboring antigens and naïve T cells, leading to co-localization and T cell priming (230). For TB, the association of iBALT formation with protective phenotypes in human lung tissue as well as in the mouse and non-human primate models point towards a protective feature against active disease (93, 213, 231, 232).

In TB, the extent of T and B cell priming in the iBALT versus lung-draining lymph nodes has been incompletely explored. The priming of naïve antigen specific CD8 T cells after non-replicating viral vector infection has been demonstrated in adoptive cell transfer models in mice (230). However, in the mouse model of TB, priming of naïve T cells in the lung is undetectable (178, 192, 233). Memory T cell priming in the lungs has only been shown after the intratracheal transfer of antigen-laden dendritic cells (234). Yet previously activated T and B cells do traffick through the iBALT in other infectious models (229). Tfh cells which are critical to germinal center formation and B cell differentiation into antibody-producing plasma cells are normally found in secondary lymphoid organs lymph node and spleen and may have the same role in iBALT (107, 235). T cells that express properties of both Th1 and Th17 cells, along with Tfh cells are found in the lung, express CXCR5, produce IFNγ and/or IL17 and are associated with iBALT formation and control of *Mtb* infection (93, 107). For protection against TB, Tfh cells in the lung support germinal center responses involved in (1) the development of memory B cells and differentiation into antibody producing plasma cells, (2) the regulation of pathologic immune responses such as neutrophilia and cavity formation, (3) the classical priming of T cells locally, and (4) the non-classical activation of CD8 and CD1-restricted T cells by cross-priming and efferocytosis (130, 218, 219).

The formation of iBALT near bronchi or in the lung interstitium is influenced by the infecting microbe and host response (236, 237). The recruitment of dendritic cells, macrophages, and innate lymphoid cells leads to cytokine and chemokine production that enlists B and T cells (223, 230). More specifically, in early *Mtb* infection, type 3 innate lymphoid cells (ILC3s) (223) and production of IL23, IL17, IL22 and CXCL13 direct B and T cells into the lung of the mouse model (238). Within the iBALT, conventional dendritic cells and T cells localize around B cell follicles into germinal center-like structure. Over time distinct T cell and B cell zones are formed (228, 230). Thus, lymphocytes expressing CCR7 and CXCR5 traffick to and organize the iBALT (93, 239). In addition, CCL19, CCL21 and CXCL13 from follicular dendritic cells are critical to establishing structure (228). These same axes (CCR7-CCL19/CCL21 and CXCL13-CXCR5) are required for T and B cell homing, antigen presentation, T cell activation, IFNγ production and control of *Mtb* growth (93, 222, 240). Finally, a recent transposon mutagenesis screen in the non-human primate model of TB identified gene associated with production of *Mtb* cell wall lipids with the formation of iBALT (98). Together, these studies show that the extent of protection offered by the iBALT (241) is likely dependent on both the infecting organism and the host.

## Complexity of Immunity in TB

### Protection and Sterilizing Immunity

One goal of an effective TB vaccine is to prevent infection or provide sterilizing immunity to the host. However, generating a sufficiently robust and durable immune response that neutralizes and eradicates *Mtb* could come at a survival cost to the host. To this point, enhancement of CD4 T cell responses at the level of immune checkpoints such as PD-1 counterintuitively enhances disease and decreases survival in mouse studies (242). In some patients, anti-PD-1 monoclonal antibodies used to treat cancer precipitated reactivation TB from latently infected individuals (242). Indeed, *post-hoc* analyses of the MVA85A clinical trials demonstrate that higher frequency of HLA-DR+ activated CD4 T cells can be associated with increased risk of TB while higher Ag85A IgG titers correlate with protection (243). Thus, strategies that release the break on T cells unilaterally could provide more harm than benefit. Nevertheless, a cytomegalovirus vectored vaccine generating antigen-specific T cell but no antibody responses prevented TB in nearly half of the *Mtb*-challenged non-human primates (244). Elucidating how immune responses can provide benefit with minimal cost will likely have broad applicability to vaccines across many infectious diseases.

Most TB vaccines in clinical development now incorporate both antibody and T cell responses, though which are correlates of protection and which of disease remain to be fully clarified. These vaccines include but are not limited to the M72-AS01e subunit vaccine containing a recombinant fusion protein of Mtb32a and Mtb39a (245) and BCG in multiple forms (246). While much has been evaluated involving the classic intradermal delivery, revaccination (247, 248) and the use of the recombinant VPM1002 expressing listerolysin-O to enhance CD8 T cell responses (249, 250) are variations currently in clinical trials. In pre-clinical models there is attention paid to delivery route- intravenous (32, 251), intranasal, aerosol, intratracheal and endobronchial (252). For both BCG and MTBVAC, an attenuated *Mtb* strain lacking the phoP and fadD26 virulence factors, antigen specific IgG are detected in pulmonary but not intradermal vaccinated animals and facilitate *Mtb* opsonophagocytosis *in vitro* (253). Thus, independent of what is induced during natural infection and disease, vaccine strategies that incorporate a greater breadth and depth of responses including B cells, antibodies and trained innate immunity (254, 255) could complement T cell responses to more effectively eradicate *Mtb* (32, 251, 256, 257).

### Pathology and Disease

A second goal of an effective vaccine is to prevent TB disease and lung pathology. This, notably, does not necessarily imply eradication of the bacteria. In humans, there is increasing recognition that latent TB may represent a protective state (2, 258). Indeed only 5-10% of individuals progress to active disease. Yet the immune mechanisms by which the transition between latent and active disease occurs in humans and the corollary of how the remaining 90-95% of individuals maintain the asymptomatic latent state – whether this be with dormant or eradicated bacteria (2) – are not known. This phenotype has been difficult to study in animal models. The classic murine model recapitulates active TB but not latent infection. Furthermore, there is a disconnect between *Mtb* burden, dissemination and survival observed in passive antibody transfer studies of mice (259, 260). Thus, mechanisms by which asymptomatic *Mtb* latent infection is sustainably induced could be leveraged for vaccine design. To this point, antibodies have the potential to enhance disease whether directly through increased replication such as in Dengue hemorrhagic fever and *Leishmania* or indirectly through inducing a dysregulated immune response such as in COVID-19 (261). Understanding whether and how antibodies can enhance TB disease by mediating pathological inflammation in immune reconstitution inflammatory syndrome or simply supporting dissemination (262, 263) could provide novel host directed therapeutic targets.

Several candidate subunit TB vaccines aim to prevent not only initial infection but also pathology once infection has occurred. These include, but are not limited, to H56:IC31 that encompasses Ag85B, ESAT6 and Rv2660c (264), and the ID93 + GLA-SE vaccine which contains a TLR4 agonist formulated with a recombinant fusion protein of the *Mtb* virulence factors (esxV/Rv3619, esxW/Rv3620, PPE42/Rv2608) and *Mtb* latency associated protein Rv1813 (256, 265, 266). Administration of these vaccines in animal models induces robust antigen specific immune responses that, when combined with antibiotics, is linked to decreased bacterial burden and lung pathology in comparison to antibiotics alone. The highly effective mRNA vaccines BNT162b2 and mRNA-1273 successfully prevents both infection and serious disease with spike specific T cell and antibody responses against SARS-CoV2 (267, 268). As such, mRNA platforms provide promising avenues for TB vaccines in generating immune responses that both protect against *Mtb* infection and prevent disease pathology.

### Challenges in Modeling Tuberculosis

Inherent differences between the widely used inbred mouse models and humans are critical to acknowledge when extrapolating data (269). Antibody translocation and recycling functions of FcRN and pIGR in humans are recapitulated in the murine model. Similarly, the non-classical IgG binding TRIM21, the single IgM receptor (FcμR), the two receptors for IgM and IgA (pIGR and Fcα/μR/CD351) and the two receptors that bind to IgE (FcεRI and FcεRII) in humans are also found in mice. Unique to humans is FcαRI/CD89 which binds only IgA. For the classical IgG receptors (24), the expression of the activating FcγRI/CD64 in mice is limited to monocytes, macrophages and dendritic cells whereas in humans the spectrum also includes neutrophils. Conversely, FcγRIIIa/CD16 in humans is restricted to natural killer cells, monocytes and macrophages while in mice the spectrum also includes neutrophils, dendritic cells, basophils, eosinophils and mast cells. The only inhibitory Fc receptor, FcγRIIb/CD32b, is detected primarily on B cells and other myeloid lineage cells in mice but in a more limited capacity in humans. The activating FcγRIIa/CD32a, FcγRIIc/CD32c and FcγRIIIb/CD16b exist exclusively in humans while FcγRIV/CD16.2 is unique to mice. In addition, there is no clear equivalence between the FcR binding capacity of the human subclasses IgG1, 2, 3 and 4 and mouse IgG1, 2a/c, 2b and 3. Finally, the mouse IgG glycome partially overlaps with that of humans, with the functional implications of the differences not yet defined (36, 270). Mice with human FcR and signaling adaptors (271) as well as immunoglobulin genes (272) provide paths towards bridging the species gap. However, ensuring similarities in expression patterns to recapitulate immune effector functions at baseline and during infection remains to be fully flushed out as levels of each receptor influence each other.

For TB, the widely used low dose aerosol inbred mouse model does not recapitulate latent infection and poorly captures the phenotypic heterogeneity observed in humans (9, 273). The absence of some mechanisms of protection involved in latency may be one reason why results from vaccines studies in mice do not always directly translate to humans. For example, the promising 1-2 fold log reduction in colony forming units in the mouse model with BCG is reflected by reduction of disseminated TB in the pediatric population and variable impact on adult pulmonary TB, the most common form (274). Whether the aforementioned differences in antibody responses between mice and humans could be a factor in this gap is not known. Questions to this point could be addressed with diverse outbred mice and or new models of TB such as ultra-low dose aerosol infection that have the potential to reflect latent infection more closely than the current most widely used approach (275–277). Nevertheless, the canonical (and tractable) inbred mouse model has generated data that has formed the foundations of our understanding of antibody Fc effector functions in autoimmunity and monoclonal antibody fields. Building on this knowledge to address specific questions in the context of *Mtb* that could be orthogonally tested in non-human primates and or *ex vivo* work with patient samples provide paths to dissect physiologically relevant mechanisms of disease.

### Challenges in Defining Human TB

The development and exploitation of robust imaging, microbiology and immune correlates techniques to characterize the spectrum of TB observed clinically has transformed the classic framework of uninfected, latent infection and active disease. Now it is possible to begin to better identify individuals with high exposure, initial infection, the quiescent bacterial state, subclinical infection, chronic smoldering disease that progresses and regresses, overt active disease, bacterial persistence after treatment and bacterial cure (41, 278–283).

Beyond pulmonary TB, extrapulmonary disease involving hematogenous dissemination to the central nervous system, bone marrow and other organs characterize a subset of cases. The risk of multiorgan involvement increases with immunosuppression. However, the precise mechanisms by which these additional TB states develop are largely unknown but likely involve both bacterial and host protective and pathologic immune factors (284).

Notably, not all immunosuppression leads to TB. Over 90% of TST+ individuals do not develop active TB after receiving anti-TNFα therapy, solid organ or hematopoietic stem cell transplantation (2). Clinical observations in heavily immunosuppressed adults such as these, particularly in the absence of routine antibiotic prophylaxis for TB, indicate that a proportion had elicited sterilizing immunity and cleared the infection. In neonates, accidental delivery of live virulent *Mtb* contaminating the BCG vaccine in the pre-antibiotic era led to death in only 29% of the cases and the surviving 70% had significant variation in clinical phenotypes (285). These observations demonstrate that there are likely multiple paths to protection that leverage a diverse array of innate and adaptive immune responses. Thus, in addition to CD4 T cell production of IFNγ, the distinct presence of B cells in granulomas in the *Mtb* infected lung as well as B cell follicles and germinal centers in the adjacent iBALT raise the possibility that their functions influence outcomes. Similarly, the presence of antibodies amidst the FcR bearing monocytes, macrophages, neutrophils, dendritic cells and T cells recruited to an *Mtb* lesion points towards Fab and Fc domain mediated effector functions that locally coordinate host responses to bacteria. In revisiting the paradigm of protection and disease, linking diverse immune responses that include antibodies, B and T cells will enable the re-examination of the heterogenous spectrum of human TB.

## Author Contributions

LL and SC drafted and wrote the manuscript, and approved the submitted version.

## Funding

SC is supported by University Hospitals Cleveland Medical Center. LL is supported by the National Institutes of Health under Award Number R01AI158858 and the Disease Oriented Scholars Award at UT Southwestern Medical School.

## Conflict of Interest

The authors declare that the research was conducted in the absence of any commercial or financial relationships that could be construed as a potential conflict of interest.

## Publisher’s Note

All claims expressed in this article are solely those of the authors and do not necessarily represent those of their affiliated organizations, or those of the publisher, the editors and the reviewers. Any product that may be evaluated in this article, or claim that may be made by its manufacturer, is not guaranteed or endorsed by the publisher.
